# Rebound-Hyperkalzämie nach Denosumab-Therapie bei einem 13-jährigen Jungen mit aneurysmatischer Knochenzyste des Humerus – ein Fallbericht

**DOI:** 10.1007/s00132-025-04745-1

**Published:** 2025-11-25

**Authors:** Beatrice Jung, Frank Traub, Tilmann Busse, Eren Demir

**Affiliations:** Zentrum für Orthopädie und Unfallchirurgie, Universitätsmedizin Mainz, Langenbeckstr. 1, 55131 Mainz, Deutschland

**Keywords:** Knochenneoplasien, Kinder, Calcium aus der Ernährung, Humerus, Off-Label-Therapie, Bone neoplasms, Children, Dietary calcium, Humerus, Off-label use

## Abstract

Denosumab ist für die Therapie der Osteoporose und ausgewählter Knochentumoren zugelassen, und seine Wirksamkeit ist in der Literatur gut dokumentiert. Bei Kindern erfolgt der Einsatz zur Behandlung benigner Knochentumoren jedoch off-label. Wir berichten über einen 13-jährigen Jungen mit aneurysmatischer Knochenzyste des Humerus, der nach Beendigung einer supportiven Denosumab-Therapie eine ausgeprägte Hyperkalzämie entwickelte. Der Patient wurde erfolgreich mit intravenöser Flüssigkeitstherapie, Kaliumsubstitution, Zoledronat und Furosemid behandelt. Dieser Fall verdeutlicht die Notwendigkeit einer engmaschigen Kontrolle des Calcium- und Knochenstoffwechsels bei Kindern und Jugendlichen während und nach Denosumab-Therapie.

## Abstract (en)

Denosumab is approved for the treatment of osteoporosis and selected bone tumors, with its efficacy well documented in the literature. However, its use in children for benign bone tumors remains off-label. We report the case of a 13-year-old boy with an aneurysmal bone cyst of the humerus who developed severe hypercalcemia following the cessation of supportive denosumab therapy. The patient was successfully treated with intravenous fluid therapy, potassium supplementation, zoledronate, and furosemide. This case highlights the necessity for close monitoring of calcium and bone metabolism in children and adolescents during and after denosumab treatment.

## Anamnese

**Abb. 1:**
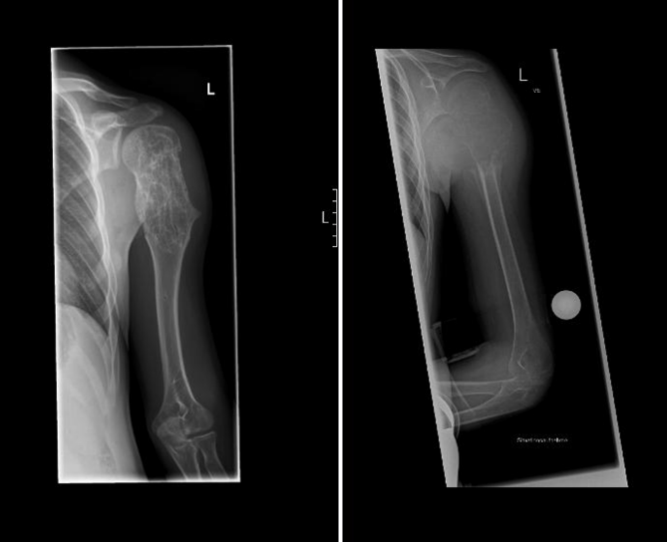
Verlaufsdokumentation der linken oberen Extremität: *rechts* die Ausgangssituation 2022 mit aneurysmatischer Knochenzyste vor En-bloc-Resektion; *links* die Situation nach Therapieabschluss im Oktober 2025, nach En-bloc-Resektion, Rekonstruktion mittels nicht vaskularisierter Fibula, ESIN-Implantation, Polidocanol-Instillationen und Denosumab-Gabe, mit deutlicher Rückbildung der zystischen Läsionen und Regeneration der Knochenstruktur

Bei einem damals 11-jährigen Jungen wurde im Dezember 2021 in einem externen Krankenhaus eine pathologische Fraktur des linken Humerus diagnostiziert und zunächst dort behandelt (Abb. 1). Die Röntgenaufnahme zeigte eine zystische Struktur im proximalen Humerus. Im Februar 2022 erfolgte im selben Haus eine MRT-Untersuchung, die eine aneurysmatische Knochenzyste mit Destruktion des Humeruskopfes sowie der Epiphysenfuge bis hin zur Metaphyse offenbarte. Im März 2022 erfolgte zunächst die histologische Sicherung, anschließend eine En-bloc-Resektion mit Rekonstruktion mittels nicht vaskularisierter Fibula, die durch eine elastisch-stabilisierende intramedulläre Nagelung (ESIN) fixiert wurde. Eine adjuvante oder neoadjuvante supportiv-therapeutische Maßnahme wurde nicht eingeleitet.

Im August 2022 wurde der Patient erstmals in unserer Abteilung vorgestellt. Zu diesem Zeitpunkt wurde eine erneute Kürettage mit Instillation von Polidocanol geplant sowie eine supportive Therapie mit Prolia (Denosumab) festgelegt.

Während der Behandlung in unserer Klinik wurden insgesamt drei Kürettagen unterschiedlicher Zystenanteile mit anschließender Instillation von Polidocanol 3 % durchgeführt; zusätzlich erhielt der Patient innerhalb von 8 Monaten vier subkutane Gaben von Denosumab, um das Fortschreiten der aneurysmatischen Knochenzyste bis zum Abschluss des Wachstums zu unterdrücken.

Zum damaligen Zeitpunkt war Denosumab für Kinder und Jugendliche nicht zugelassen; verfügbare Studien und Zulassungen bezogen sich überwiegend auf erwachsene Patienten, insbesondere bei Osteoporose, Knochentumoren oder multiplen Myelomen.

Im Juli 2023 wurden die ESIN entfernt, und in derselben Operation erfolgten eine Instillation von Polidocanol 3 % sowie eine lokal begrenzte Kürettage einzelner verbliebener zystischer Anteile. Sechzehn Wochen nach der letzten Denosumab-Gabe stellte sich der inzwischen 13-jährige Junge mit wiederholten Beschwerden in der heimatnahen Notaufnahme vor, darunter Bauchschmerzen und Schmerzen in beiden Knien.

## Befund

Der klinische Aufnahmestatus zeigte einen kraftlosen, schmerzgeplagten 13-jährigen Jungen mit passiv frei beweglichen Gelenken und ohne Hinweise auf Infektion. Er klagte über diffuse Bauchschmerzen.

## Aufnahmelabor

Laborchemisch wurde eine ausgeprägte Hyperkalzämie mit einem Kalziumspiegel von 3,8 mmol/l festgestellt. Zudem zeigte sich eine eingeschränkte Nierenfunktion mit einem Kreatininwert von 1,23 mg/dl und erhöhtem Harnstoff. Phosphat sowie alkalische Phosphatase lagen im Normbereich. Ein Hyperparathyreoidismus, Vitamin-D-Überdosierung sowie Hypothyreose konnten ausgeschlossen werden. Die Urindiagnostik ergab keine Hinweise auf verminderte Ausscheidung von Calcium oder Phosphat.

## Apparative Diagnostik

Aufgrund wiederholter Schmerzen in beiden Knien und unspezifischer Bauchbeschwerden sowie der bekannten Anamnese einer aneurysmatischen Knochenzyste wurde bei Aufnahme eine Ganzkörper-MRT durchgeführt. Diese sollte mögliche weitere Knochenbeteiligungen, metabolische Knochenveränderungen oder frühzeitige Komplikationen identifizieren.

**Abb. 2:**
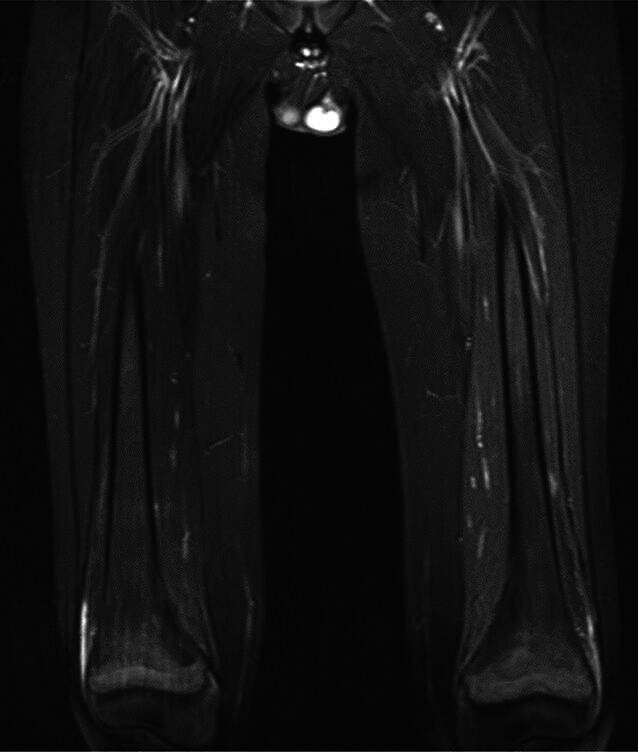
Bilaterale Knochenödeme in den distalen Metaphysen der Femora

**Abb. 3:**
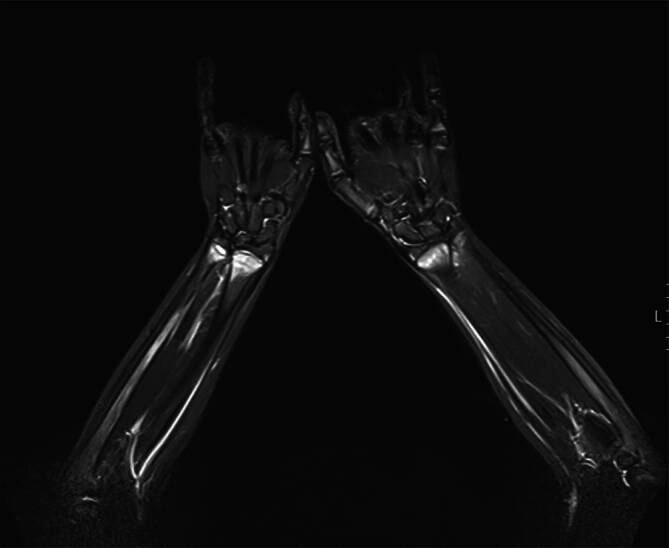
Bilaterale Knochenödeme in den Epi- und Metaphysen von Ulna und Radius beidseits

**Abb. 4:**
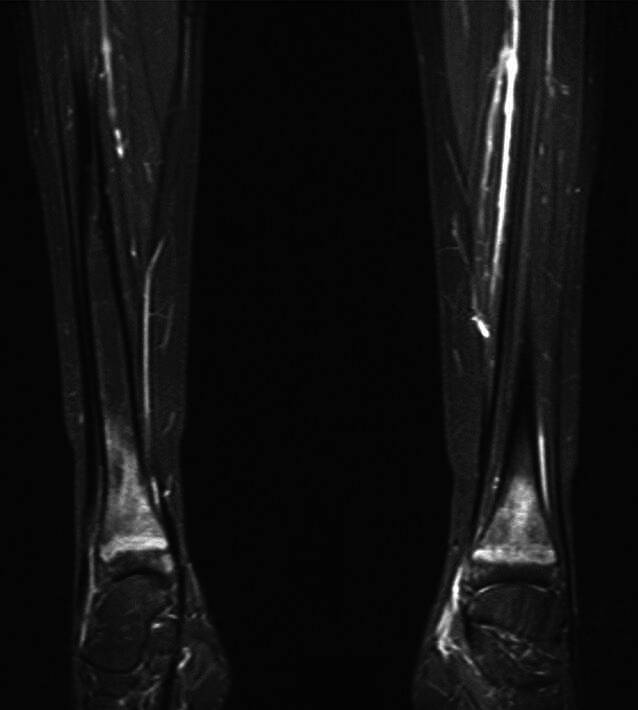
Bilaterale Knochenödeme in den distalen Metaphysen von Tibiae und Fibulae

**Abb. 5:**
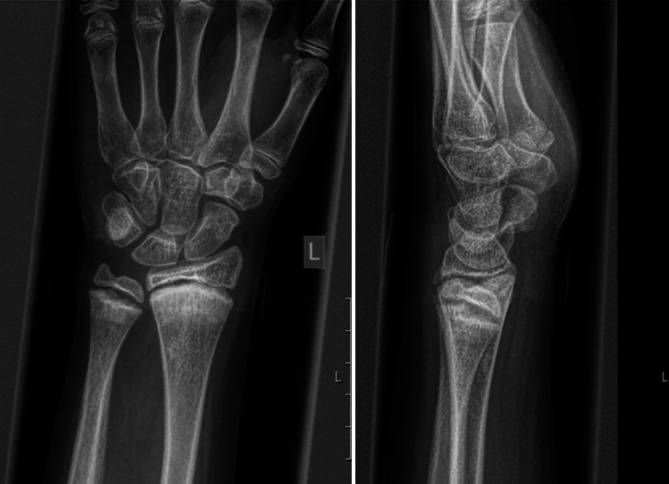
Flächige Sklerosierung der distalen Metaphysen von Radius und Ulna beidseits

In der MRT zeigten sich bilaterale Knochenödeme in den distalen Metaphysen der Femora (Abb. 2), Radii (Abb. 3), Ulnae, Tibiae und Fibulae (Abb. 4) sowie im rechten proximalen Humerus. Diese Ödeme spiegeln eine reaktive Knochenumbauaktivität insbesondere in den Wachstumsfugen wider. Dabei handelt es sich um eine überschießende Osteoklastenaktivität mit konsekutiver erhöhter Knochenresorption nach abruptem Wegfall der RANKL-Inhibition. Pathophysiologisch liegt eine reaktive Aktivierung akkumulierter Osteoklastenvorläufer zugrunde, klinisch manifestierend insbesondere bei jungen Patienten mit einer Hyperkalzämie durch gesteigerte Calciumfreisetzung. Das ergänzende Röntgenbild der Hand (Abb. 5) offenbarte eine flächige Sklerosierung der distalen Metaphysen von Radius und Ulna. Die Sonographie des Abdomens zeigte eine Renomegalie mit Nephrokalzinose Grad 2b. Das EKG war altersentsprechend unauffällig.

## Diagnose

Klinisch, laborchemisch und radiologisch zeigten sich die Befunde einer Hyperkalzämie nach Beendigung einer Denosumab-Therapie. Laborchemisch bestand eine deutlich erhöhte Kalziumkonzentration bei gleichzeitig eingeschränkter Nierenfunktion. Bildgebend zeigte sich ein ausgedehntes Knochenödem in mehreren Metaphysen der langen Röhrenknochen sowie eine flächige Sklerosierung im Bereich des distalen Radius und der Ulna. Sonographisch fanden sich eine beidseitige Nierenvergrößerung und eine Nephrokalzinose Grad 2b. Das EKG war unauffällig.

Diese Befunde wurden im klinischen Kontext als Rebound-Hyperkalzämie nach Denosumab-Therapie gewertet.

## Therapie und Verlauf

Bei Entwicklung einer Hypokaliämie wurde zunächst eine orale Substitution durchgeführt, die aufgrund von Unverträglichkeiten intravenös fortgesetzt wurde. Die Flüssigkeitstherapie erfolgte mit Natriumchlorid 0,9 % und Kaliumzusatz bei einer Rate von 120 ml/h. Zur Senkung des Calciumspiegels wurde einmalig Zoledronat (0,05 mg/kg) verabreicht, wodurch vermehrt Calcium in den Knochen eingebaut wurde.

Furosemid (3 × 5 mg) wurde zur Förderung der Calciumausscheidung eingesetzt, angepasst an den fallenden Calciumspiegel. Die tägliche Flüssigkeitsmenge (oral und intravenös) betrug insgesamt 3000 ml. Unterstützend wurde eine kalziumarme Diät etabliert.

**Abb. 6:**
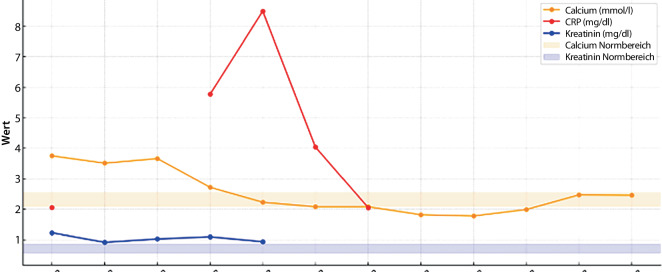
Graphische Darstellung des zeitlichen Verlaufs des Serum-Calciumspiegels sowie der Nierenfunktionsparameter über 8 Tage; *CRP* C-reaktives Protein

Durch diese Maßnahmen normalisierte sich der Calciumspiegel innerhalb von 8 Tagen, die Nierenfunktionsparameter lagen bereits nach 4 Tagen wieder im Normbereich (Abb. 6). Der Patient blieb kardiopulmonal unauffällig, und die Vitalparameter wurden kontinuierlich überwacht. Interdisziplinäre Betreuung durch Physiotherapie, Ergotherapie, Psychologie und Ernährungsberatung unterstützte die Rehabilitation. Nach 12 Tagen wurde der Patient entlassen; die Calciumwerte blieben bei Nachkontrollen stabil, und die Symptome bildeten sich vollständig zurück.

## Diskussion

Denosumab ist für Kinder und Jugendliche nicht zugelassen; die Anwendung erfolgt off-label, z. B. bei fibröser Dysplasie, aneurysmatischen Knochenzysten oder Osteogenesis imperfecta.

Der RANKL-Antikörper hemmt die Reifung der Osteoklasten und reduziert somit den Knochenabbau. Dadurch steigt die Knochendichte und die Stabilität, besonders im Bereich der Wachstumsfugen.

Im vorliegenden Fall entwickelte der Patient trotz geplanter, limitierter Gabe von vier Dosen über 8 Monate eine Hyperkalzämie. Die beobachtete Hyperkalzämie ist am ehesten Ausdruck eines Denosumab-assoziierten Rebound-Phänomens infolge der reaktiven Osteoklastenaktivierung nach Wegfall der RANKL-Inhibition.

Diese systemische Reaktion wird bei pädiatrischen Patienten durch die physiologisch gesteigerte Knochenremodellierung in der Wachstumsphase zusätzlich verstärkt.

Im vorliegenden Fall könnte die mineralisierte Matrix innerhalb der aneurysmatischen Knochenzyste als lokaler Calcium-Pool fungiert haben, dessen rasche Resorption nach Wiederanspringen der Osteoklasten zur ausgeprägten Hyperkalzämie beigetragen hat. Radiologisch zeigte sich dies als flächige Sklerosierung im Unterarm. Die beobachteten Knochenödeme in den Metaphysen spiegeln eine reaktive Umbauaktivität wider, ausgelöst durch die erhöhte lokale Knochenstoffwechselaktivität und den metabolischen Stress.

Prinzipiell stellt Denosumab auch im Kindesalter eine potenzielle Therapieoption bei Knochentumoren dar. Das lebensbedrohliche Rebound-Phänomen ist jedoch insbesondere im Kindes- und Jugendalter relevant. Regelmäßige Laborüberwachung von Calcium- und Knochenstoffwechsel während und nach der Therapie ist essenziell.

## Schlussfolgerung

Dieser Fall verdeutlicht, dass Denosumab bei Kindern und Jugendlichen zwar pathologische Frakturen wirksam verhindern kann, gleichzeitig aber das Risiko einer potenziell lebensbedrohlichen Hyperkalzämie nach Therapieende besteht – selbst bei geplanter, limitierter Gabe.

Engmaschige Überwachung des Calcium- und Knochenstoffwechsels sowie interdisziplinäre Betreuung sind unverzichtbar. Bei Off-label-Anwendungen sollte das Risiko metabolischer Komplikationen sorgfältig gegen den therapeutischen Nutzen abgewogen werden. Präventive Strategien, wie die zeitlich gestaffelte Gabe von Bisphosphonaten oder frühzeitige Laborüberwachung, verdienen besondere Berücksichtigung.

Kernaussage: Kinder und Jugendliche können von Denosumab profitieren, doch die Therapie erfordert ein hohes Maß an klinischer Vigilanz, um schwerwiegende metabolische Komplikationen frühzeitig zu erkennen und zu verhindern.
